# Is There Still Bullying in Medicine at All Levels – Undergraduate and Postgraduate? [Response to Letter]

**DOI:** 10.2147/AMEP.S311159

**Published:** 2021-03-29

**Authors:** Simon D Taylor-Robinson, Paulo Alberto De Souza Lopes, Jey Zdravkov, Rachel Harrison

**Affiliations:** 1 Department of Surgery and Cancer, Imperial College London, London, UK; 2 Department of Medicine, UAI Universidad Abierta Interamericana, Buenos Aires, Argentina; 3 Dean Street Sexual Health Clinic, London, UK; 4 Department of South East Asian Studies, School of Oriental and African Studies, London, UK

## Dear editor

We thank Sharma and her co-authors for their very insightful comments on our perspective on bullying in Medicine.^1^,^2^

We completely agree with them that bullying has been an issue at all stages in the medical career pathway, including at an undergraduate level.^1^ We agree also that to be fully comprehensive, discussion of the medical student experience should have been included, but our article went through several journals before it was accepted and the advice consistently given was to concentrate on the specific experience of our “reportee”.^2^

To this end, we did not specifically cover the medical student experience, because our report was written as a “personal perspective” of direct conversations that were had with a senior medical academic, who chose to share his experiences only as a qualified doctor and who did not talk about his experiences as a medical student.^2^ However, in our article, we did reference two studies from 2015 and 2020 which clearly demonstrate that bullying is something experienced very widely at the undergraduate level by a large proportion of medical students, as Sharma and co-authors have otherwise noted.^3^,^4^

We note and welcome the safeguarding changes made to the undergraduate experience in many countries highlighted by Sharma and her co-authors, in the creation of both personal and pastoral tutors to provide pathways to channel concerns and difficulties on all aspects of medical student welfare, including bullying.^5^

We maintain that bullying of whatever form should have no place in the Medical Profession and agree with Sharma and her co-authors that this must also involve medical student education. There can be no room for teaching by humiliation.^3^

We apologise if there has been any misunderstanding, but with respect to institutionalised bullying, we did not intend to be critical of centralised monitoring systems, such as Athena SWAN in the United Kingdom, which are designed to reward academic and medical institutions for positive steps to introduce equality and mitigate bullying.^6^ However, there is heavy emphasis on the reports generated by the institutions themselves, which in our opinion may be written to show them off in the best light. We were merely suggesting that allowing a greater emphasis on individual viewpoints (perhaps anonymised), may facilitate a channel for real life experience to be acknowledged and in so doing, bringing a 360 degree experience to the thought process on eliminating the problem from the profession. We hoped to open up a debate on the issue and are grateful to Sharma and her co-authors for their thoughtful comments.
